# A Compact Wideband Millimeter-Wave Crossover for Phased Array Antenna Systems in Remote Sensing Applications

**DOI:** 10.3390/s25123641

**Published:** 2025-06-10

**Authors:** Fayyadh H. Ahmed, Rola Saad, Salam K. Khamas

**Affiliations:** Electromagnetics, Wireless Hardware & RF Devices Group, School of Electronic and Electrical Engineering, University of Sheffield, Sheffield S1 3JD, UK; r.saad@sheffield.ac.uk (R.S.); s.khamas@sheffield.ac.uk (S.K.K.)

**Keywords:** crossover, microstrip-to-coplanar waveguide transition, millimeter wave, phase array antenna, remote sensing

## Abstract

A new compact, wideband, millimeter-wave microstrip crossover—designed without vias—demonstrates effective performance with an insertion loss of 2 dB across a wide frequency range. For Path 1, the operational bandwidth spans 11 GHz (13–24 GHz), while for Path 2, it extends over 10 GHz (12–22 GHz). The overlapping bandwidth, maintaining the 2 dB insertion loss criterion, covers 9 GHz (13–22 GHz). The design introduces two transition mechanisms to achieve optimal scattering parameters for the crossover: a stair-shaped microstrip line (MST) to ground-backed coplanar waveguide (GCPW) for the initial crossed line (Path 1), and vertical coupling between microstrip and coplanar hourglass microstrip patches on a single-layer substrate for Path 2. This innovative approach ensures an insertion loss of approximately 1 dB for both paths across the bandwidth, with a slight increase beyond 20 GHz for Path 2 due to substrate losses. Both crossed lines maintain a return loss of 10 dB across the spectrum, with isolation of approximately 20 dB. This design presents a flat, compact, and via-less configuration, with physical dimensions measuring 6.5 mm × 7.6 mm. The proposed design exhibits excellent scattering parameters, which enhance the efficiency of phased array antenna systems in terms of power transfer between input and output ports, as well as improving isolation between different input ports in the feed network of these systems used in remote sensing. Consequently, this contributes to the increased sensitivity and accuracy of such systems.

## 1. Introduction

Microwave and millimeter-wave integrated circuits are advancing rapidly and growing in intricacy. Crossovers serve as the components responsible for transmitting signals along traces that intersect physically [1]. Ensuring the necessary signal isolation is crucial for crossovers while they transmit signals. These crossovers are frequently necessary in monolithic microwave circuits, particularly within multi-channel systems, and they serve various purposes in filters, mixers, and Butler matrices for antenna array beamforming systems [2]. Phased array antennas offer a promising solution to overcoming the limitations of conventional remote sensing radiometers. Their ability to electronically steer beams without mechanical movement allows for higher spatial resolution, improved accuracy, and the capability to operate in diverse weather conditions. By replacing large mechanical reflectors with electronically controlled arrays, these antennas can improve oceanographic observations by enhancing data accuracy and expanding monitoring capabilities, while also minimizing interference from land-based contaminants near coastal areas [3]. Furthermore, phased array antennas contribute to more-compact, lightweight, and adaptable remote sensing instruments. The continued development of these technologies aligns with the objectives of future Earth observation tasks, aiming for better resolution, sensitivity, and global coverage. Their implementation in future missions will significantly improve climate monitoring, meteorology, and resource management, making them a key advancement in the field of passive remote sensing [3].

Passive beamforming circuits are preferred over their active counterparts in switched beamforming systems due to their simplicity, low power consumption, and cost-effectiveness, as demonstrated by matrices such as Butler, Blass, and Nolen [4]. Among these, the Butler matrix is particularly advantageous, as it requires the fewest passive components, making it the most suitable network for passive switched-beam antennas [5].

The conventional Butler matrix typically consists of 3 dB branch-line couplers (BLCs), crossovers formed by two cascaded BLCs, and phase shifters. The size of these components primarily depends on the guided wavelength (λg). As a result, at lower frequencies, the BLC occupies a significant area on the host device board, leading to an overall increase in size [6]. Additionally, the conventional crossover not only expands the feeding network considerably but also introduces drawbacks, such as increased insertion loss and reduced sensitivity in the intended application. Reducing insertion loss in phased array antenna (PAA) networks is therefore essential for sensing applications, particularly those requiring high gain and sensitivity. A critical step toward improvement involves optimizing PAA network components, such as replacing conventional crossovers with alternatives that exhibit lower insertion loss.

The air-bridge bonds, also known as wired vias [7], represent a conventional method for designing crossovers. However, this approach results in non-planar structures, escalating both complexity and fabrication expenses. Moreover, the use of wired vias exacerbates insertion loss, particularly at higher frequencies, due to the parasitic elements they introduce [8]. An alternative method involves situating two microstrip feeding lines on different layers, yet this approach remains incompatible with planar microstrip circuits [9]. To address these limitations, various techniques such as cascaded couplers and ring couplers have been developed, as in [10,11,12]. However, these structures are constrained by their limited bandwidth, large size, and rather high insertion loss, presenting a significant challenge in modern high-capacity communication systems. Some other designs attempted to overcome the narrow-bandwidth limitation associated with the single-layer planar crossover. However, most of them utilized vias in their designs, which in turn increased the complexity of the device and the manufacturing costs [1,13,14,15,16].

A planar microstrip crossover junction is described in [1]. The design utilized a GCPW structure incorporating vias. Two variations of transitions between the microstrip line (MSL) and the GCPW structure were integrated into a double-sided printed circuit board, yielding a bandwidth of nearly 6 GHz. In [13], a crossover was designed by using a two-layer printed circuit board. One of the path lines utilized a defected ground plane and vias to construct a signal path between two nodes. The design provided a bandwidth of 10 GHz.

Another design, based on a microstrip-to-coplanar waveguide transition, has been proposed by [14]. Although the design was able to provide a bandwidth extending from DC to 40 GHz, it contained many vias to ensure the path completion and to improve the isolation between the crossed lines. To enhance isolation between the two crossed lines, diamond-shaped slots have been inserted into the crossing area for a crossover based on a microstrip-to-coplanar waveguide transition [15]. The proposed design improved the isolation by up to 23 dB and enabled phase compensation. A number of GND vias forming a quasi-coaxial section have been suggested in a planar microstrip crossover to confine the electric field around the signal via [16]. This technique was employed to enhance impedance matching. It can be noted from the aforementioned discussion that most designs exhibit a fully planar profile but are limited to narrowband operational bandwidth. Some designs offer wideband capabilities at the expense of structural complexity, such as multi-layer configurations or containing numerous vias, which consequently increase fabrication costs or insertion loss.

Recent papers have addressed bandwidth limitations, but design challenges persist in other areas. A multi-layer vertical SIW transition microwave crossover proposed in [17] uses slot lines and vias to guide TE_10_-like modes between stacked SIW layers. It achieves orthogonal signal paths with high isolation (≥17.3 dB), low insertion loss (~2 dB), and a 28.6% fractional bandwidth. However, the need for the precise alignment of multiple dielectric layers increases manufacturing complexity and costs. Performance is also sensitive to via and slot placement, and integration with planar components may require custom interconnects, complicating testing. In [18], the common wall between two parallel waveguides is replaced by a metal grating-filled dielectric substrate, enabling continuous TE slot lines and vias to guide TE_10_-mode coupling and achieving a 21% fractional bandwidth (32.1–39.1 GHz). This approach enhances bandwidth and reduces size but poses integration challenges. Embedding the etched metal grating into a machined cavity complicates hybrid metal–PCB structures, particularly in compact or monolithic systems. Unlike SIW- or microstrip-based crossovers, it lacks scalability to planar or low-cost PCB technologies, limiting its suitability for mass-produced devices like mobile or IoT systems. A symmetrical crossover is presented in [19], consisting of a central cross and 12 line segments. Using an SIGW-based implementation, the design operates at both sub-6 GHz and Ka bands (30 GHz), achieving fractional bandwidths of 44% (2–6 GHz) and 20% at 30 GHz, respectively. However, the design exhibits frequency-dependent bandwidth shrinkage, with bandwidth decreasing from 44% at 2 GHz to 25% at 30 GHz. Additionally, an unexpected groove mode (Mode 11) emerges at higher frequencies, distorting the ideal response. Finally, in [20], a planar reconfigurable crossover using rectangular dielectric channels (RDCs) was proposed, with milled channels filled with materials of varying permittivity (air, RO4360G2, and RO3010). Using seven RDCs (one per arm) enabled a 15.8% tuning range, while twenty-one RDCs (three per arm) achieved 36.9%. However, reconfiguration requires manual material replacement, making it unsuitable for real-time applications like software-defined radios or adaptive beamformers. This manual process also limits scalability for mass production and compact integration.

This paper presents a novel millimeter-wave via-less crossover operating across a broad frequency range of more than 11 GHz (13–24 GHz). The design innovation relies on vertical coupling between a microstrip line on the top surface of a single-layer substrate and a coplanar waveguide (CPW) constructed from a defected ground plane beneath it. To achieve this coupling for one path of the crossed transmission line, four hourglass-shaped patches are employed. Additionally, a finite-width coplanar waveguide with a stair-shaped structure transition to microstrip ports on the top layer of the substrate is utilized to ensure high isolation and low insertion losses between the crossing transmission lines. The stair-shaped transition facilitates the gradual transformation of electromagnetic signals and maintains constant impedance along the transition between a CPW on one side and a microstrip transmission line (MST) on the other. The letter is structured as follows: Section 2 outlines the design procedures, while Section 3 presents an equivalent circuit model, and Section 4 demonstrates the simulation and experimental results. Finally, Section 5 offers concluding remarks.

## 2. Design of the Proposed Millimeter Crossover

**Figure 1:**
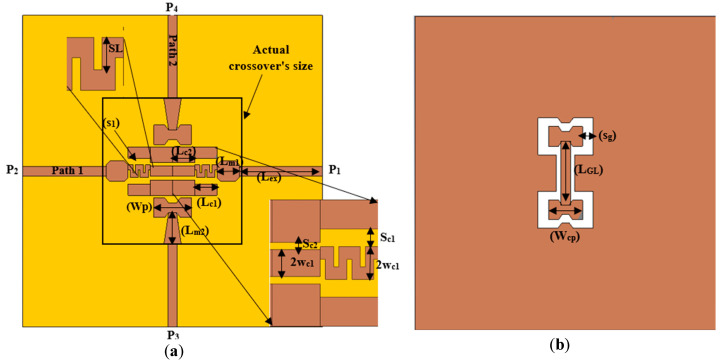
Proposed via-less crossover structure. (**a**) Top view. (**b**) Bottom view.

The proposed structure of the wideband millimeter-wave crossover is illustrated in Figure 1, with its dimensions shown in Table 1. The device can be considered an assembly of two intersecting paths, providing wideband and low insertion loss for both passing signals. Below is a description of the design details, illustrating the structure and the effect of each path.

The crossing lines connect four ports of the structure together, two ports each. Path 1, connecting port 1 and port 2, is printed on the upper side of an RC4003 Rogers substrate with a thickness of 0.406 mm. Path 1 consists of a 50 Ω microstrip line attached from one end to a ground-backed coplanar waveguide (GPWG) through a stair-shaped microstrip-to-coplanar waveguide transition in the crossing area. Path 2 connects ports 3 and 4 through a microstrip line, featuring a microstrip hourglass-shaped patch that is vertically coupled with a similar-shaped coplanar waveguide patch at their ends. The middle section is based on a CPW transmission line, constructed from the defecting substrate’s ground plane to bridge Path 1 at the crossing area, as illustrated in Figure 1b.

To delve deeper into the techniques employed in the proposed design, such as the stair-shaped coplanar waveguide transition for Path 1 and the hourglass-shaped coupled patch for Path 2, a more detailed investigation is warranted. Conducting a parametric study on these paths will facilitate a thorough examination, as outlined below.

The microstrip transmission line cannot be used in the crossing area since the ground plane of the substrate is defective there due to the creation of path 2. Instead, the ground plane should be positioned on top of the substrate to form a grounded coplanar waveguide. It is important to note that this transition from MST to GCPW introduces discontinuities for the signal along the path. Therefore, to achieve better matching results, a gradual (stair-shaped) GCPW-to-MSTL transition is employed, providing a gradual transformation of the electric and magnetic fields and maintaining almost constant impedance along the two sides of the transition [21].

**Figure 2:**
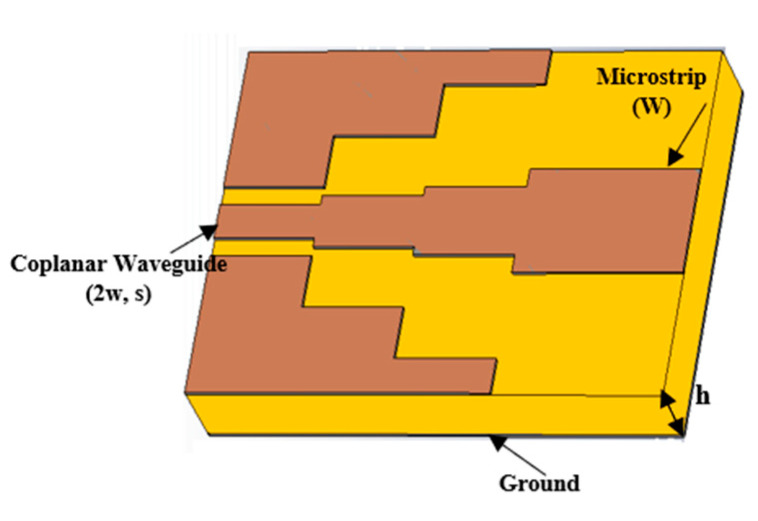
Three-stage stair-shaped microstrip line-to-coplanar waveguide line transition.

Based on the mode-matching technique, it has been shown that the characteristic impedance of the GCPW structure (as shown in Figure 2) can be considered as the parallel combination of the CPW impedance mode (Z_CPW_) and the microstrip impedance mode (Z_MS_), and these impedances are functions of the structural dimensions [22]. For a small S/h ratio, Z_CPW_ dominates; as the ratio of S/h increases, the transition structure tends to resemble a microstrip line, where it operates jointly with the ground plane underneath to guide the wave, and Z_MS_ becomes dominant. Therefore, the adopted transition is basically based on splitting the impedances, connecting the GCPW on one side to the microstrip on the other side.

In the proposed design, as illustrated in Figure 1, a stair-shaped transition of two steps, denoted as L_c1_ and L_c2_, each with a length of λg/8, is considered. The center connector of the adopted GCPW transition decreases in relation to the microstrip line width as one moves from the ports towards the crossing area. The optimum ratios in our case are 2w_c1_/Wm = 0.55 and 2w_c2_/2w_c1_ = 0.82.

Similarly, the separation between the centerline and ground lines, S, gradually decreases as we move from the microstrip side to the coplanar side, facilitating the transition of domination from the microstrip to the coplanar waveguide line. The ratio of this decrease is S_c2_/S_c1_ = 0.42.

**Figure 3:**
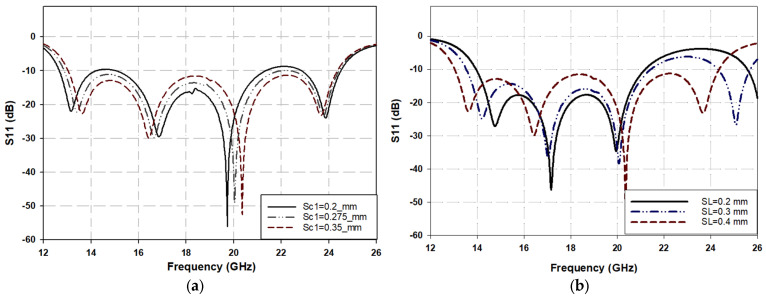
Path 1 parameters’ effect on the line impedance matching. (**a**) The first-stage gap width of the stair-shaped CPW transition gap (S_c1_). (**b**) Length of transversal slits of the center conductor of the first-stage stair-shaped CPW transition.

Changing the parameters of the first section of the stair-shaped transition (i.e., S_c1_ and SL) has a significant effect on the characteristics of Path 1. This alteration impacts the matching impedance between the microstrip impedance (Z_MS_), set at 50 Ω, and the impedance of the center part of the GCPW (Lc1 in Figure 1), Z_CPW_, in the crossing area, assumed here to be 57 Ω. It is evident from Figure 3a how decreasing Sc1 from 0.2 mm to 0.35 mm leads to a disturbance in the impedance matching (S11). This change disrupts the gradual transformation of impedances between the MST and the GCPW, causing the corresponding impedance to reduce from 57.6 Ω to 53 Ω as the separation increases from 0.2 mm to 0.35 mm.

The electrical length of Path 2 is slightly longer than that of Path 1 due to the vertical coupling (traveling) of the signal in Path 2, resulting in a slight frequency shift for the S-parameter response between the two paths. To overcome this issue, five rectangular transversal slits with dimensions of 0.2 × 0.4 mm^2^ are inserted into the center line of the first part of the stair-shaped GCPW transition (Path 1) to increase the electrical length of Path 1 while leaving the physical dimensions unchanged. Figure 3b illustrates the effect of changing the length of transversal slits on the Path 1 S-parameters, which clearly shows that decreasing the length of these slits significantly deteriorates the S-parameter, especially at the upper side of the operating band (after 20 GHz). Hence, the depth of these slits is carefully optimized to match the S-parameters of Path 1 to those of Path 2. This synthesis in tuning (impedance matching) may also be attributed to the generation of parasitic inductive elements due to these transverse slits, which in turn compensate for the value of capacitance generated by the stair-shaped discontinuity, thus achieving a wider impedance bandwidth.

In the crossover structure, Path 2 extends from port 3 to port 4, utilizing a combination of a microstrip line (L_m2_) connected to an hourglass-shaped patch on the upper side of the substrate (W_p_), facing similar-shaped slots and patches on the bottom side (W_cp_). The same combination exists at port 4, and the two combinations are connected through a coplanar waveguide line (L_GL_) created by defecting the ground plane of the structure, as depicted in Figure 1b.

In the combination, different shapes of coupled patches result in different levels of coupling; hence, it is crucial to select an aperture and patch shape that maximize coupling within a compact area to minimize spurious radiation [23]. The hourglass aperture shape stands out for its wide input impedance bandwidth and strong coupling compared to other shapes, like an H or bowtie. Moreover, its aperture size aids in reducing spurious back radiation [24]. Given these advantages, the hourglass aperture shape has been chosen for both the patch and slot in this design. The impedance matching response of Path 2 is influenced by various parameters of the electromagnetically coupled patches, including patch and aperture size, shape, and their relative positions [23].

**Figure 4:**
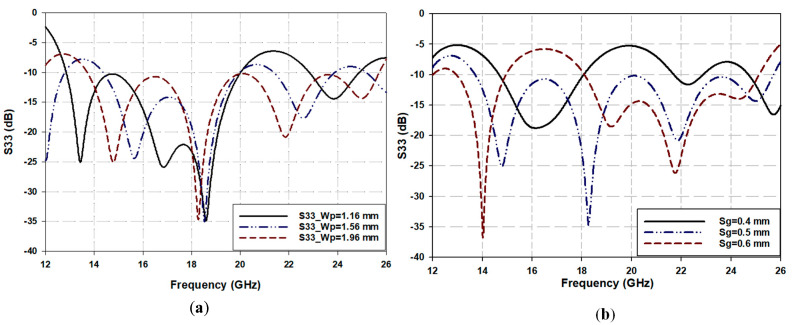
Path 2 parameters’ effect on the line impedance matching. (**a**) Microstrip W_p_. (**b**) Sg.

In the configuration of Path 2, widening the upper and lower coupled hourglass patches with the appropriate slot width of the coplanar waveguide line in the ground plane generates a tight and frequency-dependent vertical-coupled microstrip-to-coplanar waveguide transition. This coupled patch structure can be considered as a series capacitor element or a tightly coupled parallel transmission line. Broadband transmission line behavior is expected from this coupled line at the upper band end, where the length of the coupled patches is almost a quarter wavelength, $\lambda_{g}/4$. It is intuitively expected that enlarging the coupled vertical patches width (W_p_) would tighten the coupling between them, as shown in Figure 4a, as their equivalent series capacitor increases.

On the other hand, the gap between the CPW hourglass patch and the ground plane (Sg), as shown in Figure 1b, is considered a quasi-open circuit with an equivalent capacitance, Ccsg, inversely related to the size of the gap [25]. Therefore, keeping the microstrip and CPW hourglass-shaped patches unchanged, and increasing or decreasing the gap beyond a particular value will mistune the corresponding capacitance, resulting in a mismatch in the reflection coefficient of the path at different frequency ranges, as shown in Figure 4b.

**Figure 5:**
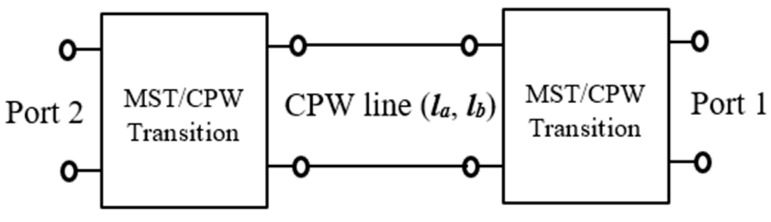
Back-to-back microstrip-to-coplanar transition structure.

The S-parameters of the vertical-coupled back-to-back microstrip to coplanar transition can be determined using the configuration depicted in Figure 5 [25]. This configuration consists of a central coplanar waveguide transmission line with two vertical-coupled MST/CPW transitions at its ends. If the central coplanar waveguides of the configuration are represented with two different lengths, *l_a_* and *l_b_*, and their corresponding S-parameter coefficients are $S_{11}^{a},S_{22}^{a},S_{21}^{a}$, $S_{12}^{a}$ and $S_{11}^{b},S_{22}^{b},S_{21}^{b}$, $S_{12}^{b}$, respectively, then the scattering parameters of the MST/CPW transition, $S_{11}^{M/C}$, $S_{22}^{M/C}$, $S_{21}^{M/C}$, can be expressed based on the two previously measured sets of scattering matrices, as follows:

(1) $$S_{11}^{M/C}=\frac{S_{11}^{a}S_{21}^{b}e^{-\gamma l_{b}}-S_{11}^{b}S_{21}^{a}e^{-\gamma l_{a}}}{S_{21}^{b}e^{-\gamma l_{b}}-S_{21}^{a}e^{-\gamma l_{a}}}$$

(2) $$S_{22}^{M/C}=\frac{S_{11}^{b}-S_{11}^{a}}{S_{21}^{b}e^{-\gamma l_{b}}-S_{21}^{a}e^{-\gamma l_{a}}}$$

(3) $${{(S}_{21}^{M/C})}^{2}=\frac{{2S}_{21}^{b}S_{21}^{a}sinh[\gamma (l_{a}-l_{b})]}{S_{21}^{b}e^{-\gamma l_{b}}-S_{21}^{a}e^{-\gamma l_{a}}}$$

## 3. The Equivalent Circuit Model

**Figure 6:**
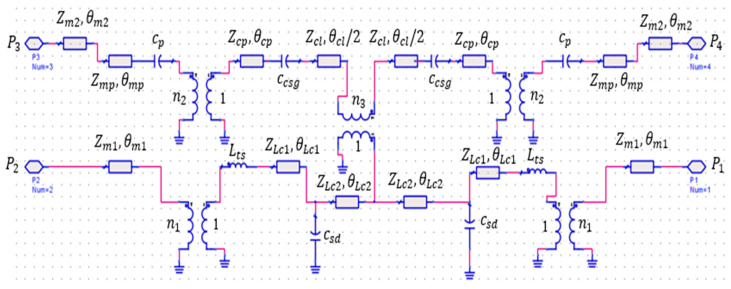
Impedance lumped equivalent circuit of the proposed crossover circuit.

The proposed device’s approximate equivalent circuit, shown in Figure 6, is derived using Keysight’s Advanced Design System (ADS) software, version ADS2016.01. It represents MST and CPW lines with impedance, Z, and electrical length, θ. MST-to-CPW transitions and line discontinuities are treated as transformers with near-unity ratio and lumped L or C elements. This circuit aids in comprehending the device’s crossover operation. In this circuit, Path 1’s equivalent circuit represents the first microstrip line (L_m1_) as a 50 Ω impedance (Z_m1_) with an electrical length (θ_m_) of λg/2. The first section of the stair-shaped CPW-to-MST transition (L_c1_) is modeled as an impedance (Z_Lc1_) of 53 Ω with a length of θ_Lc1_ = λg/8. The second section (L_c2_) is replaced by an impedance (Z_cl2_) of 57.5 Ω and an electrical length (θ_Lc2_) of λg/8.

The stair-shaped transition between the microstrip line and GCPW itself is represented by a transformer with a transformation ratio (n_1_). The coupling level between the two lines across the transition determines the value of n_1_ and is typically considered to be close to 1. In this circuit, the optimal value of n_1_ obtained from ADS optimization that closely matches the results of CST is 0.97. A series inductance, L_ts_, of 63 pH is yielded due to rectangular transversal slits in the first section of the CPW [26]. This inductance is useful in compensating for the parallel capacitance, C_sd_, of 158 fF due to discontinuities between the stair-shaped parts of the transition [26], or, in some cases, for fine-tuning the microstrip line length.

Now considering Path 2, the segments of the transmission line, θ_m2_ of λ_g_/2 and θ_mp_ of λ_g_/8, with a lumped impedance of Z_m2_ and Z_mp_ equal to 50 Ω, represent the equivalent lumped circuit for L_m2_ with the line extension L_ex_ and W_p_ of Figure 1, respectively. The capacitors C_p_ and C_csg_, with very low respective values of 26 fF and 39 fF, respectively, represent the fringe effects at the open-ended hourglass-shaped microstrip patch and CPW patch of the transition, while the transition itself is represented by the transformers with a particular transformation ratio, n_2_, close to 1. The value of n_2_ is considered as 0.97 in this equivalent circuit.

**Figure 7:**
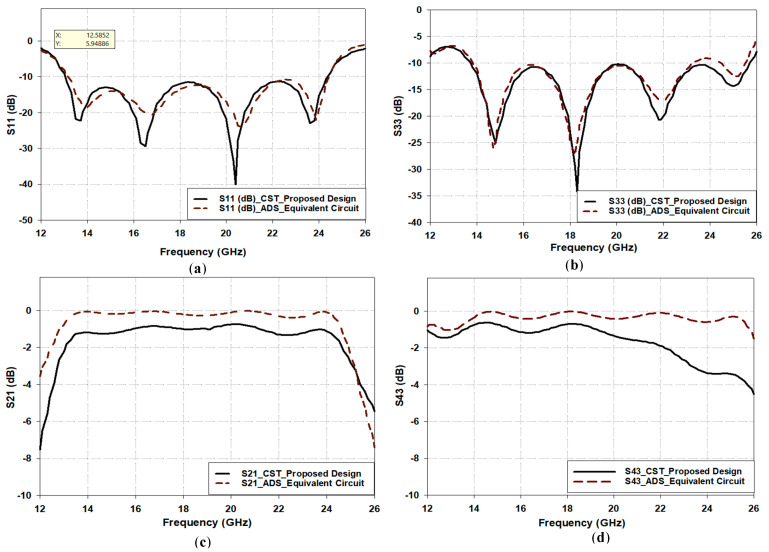
Comparison between scattering parameters of the proposed crossover in Figure 1 and its equivalent circuit in Figure 6. (**a**) Return loss of Path 1 (S11). (**b**) Return loss of Path 2 (S33). (**c**) Insertion loss of Path 1 (S21). (**d**) Insertion loss of Path 2 (S43).

The lower hourglass coplanar patch, W_cp_, and CPW line, L_GL_, of Figure 1 are chosen to be 50 Ω lumped impedance (Z_cp_ and Z_cl_) with lengths θ_cp_ and θ_cl_, equal to almost 1.5 λ_g_/8 and λ_g_/3, respectively, in the equivalent circuit diagram of Figure 6. Finally, the cross-intersection between the top and the bottom CPW on both sides of the substrate in the crossing area yields a kind of coupling between the two paths. This coupling is represented by a transformer with a transformation ratio (n_3_) connecting the equivalent circuits of the two paths. Since the coupling between the two paths must be very low, the value of n_3_ has been considered to be 0.1 in the equivalent circuit. To validate the equivalent circuit accuracy, Paths 1 and 2’s scattering parameters were studied by using the optimized equivalent circuit model using ADS and the full-structure simulation in CST, as shown in Figure 7, with close agreement. However, the equivalent circuit has a lower insertion loss than the CST simulations by 1 dB over the 13 to 25 GHz band. This is expected because the equivalent circuit does not consider the dielectric losses of the substrate as in the CST simulations.

## 4. Results and Discussion

**Figure 8:**
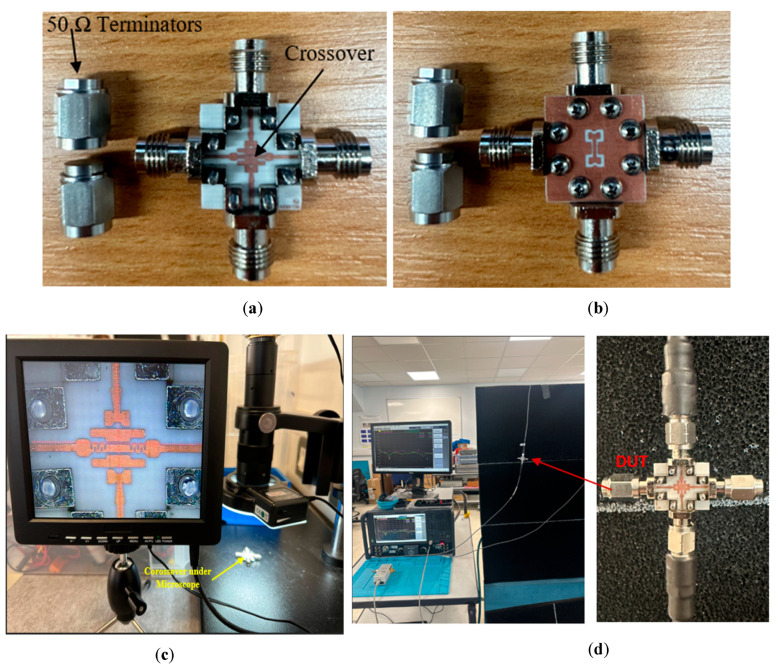
A prototype of the proposed crossover. (**a**) Front view, (**b**) back view, (**c**) prototype under a microscope, and (**d**) measurement-setup system.

The prototype of the proposed crossover is presented in Figure 8a,b, where a 0.406 mm RO4006 substrate was utilized. The actual size of the crossover structure is 6.5 × 7.6 mm^2^, whereas the overall size of the PCB is 15.24 × 16.3 mm^2^. As the proposed design features a significantly compact size, its details cannot be clearly demonstrated with a normal image. Therefore, the prototype was placed under a microscope to reveal the tiny details, particularly at the crossing area, as shown in Figure 8c. As mentioned earlier, the four ports have been extended to accommodate RF connectors. The prototype was measured using Vector Network Analyzer (Keysight, PNA-X, N524B), as shown in Figure 8d. The parameters of interest include the return and insertion losses for, as well as isolation between, the two paths: (S_11_ and S_33_), (S_21_ and S_43_), and (S_31_ and S_13_), respectively. The scattering parameters for ports 2 and 4 were not considered, as they are identical to those of ports 1 and 3.

**Figure 9:**
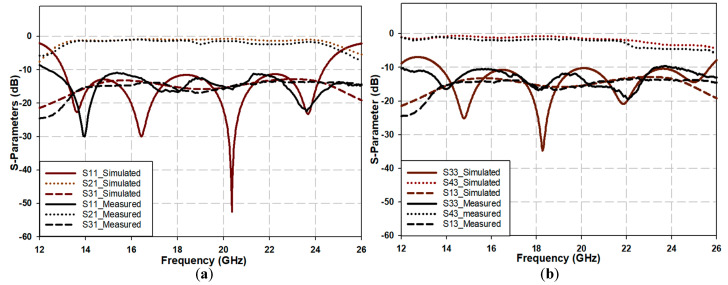
Scattering parameters of the proposed crossover. (**a**) Scattering parameters of Path 1. (**b**) Scattering parameters of Path 2.

Figure 9a,b compare simulated and measured results. Figure 9a illustrates the S_11_, S_21_, and S_31_ parameters. Meanwhile, Figure 9b illustrates the corresponding parameters for Path 2 (i.e., S_33_, S_42_, and S_13_). It is evident that the measured results closely resemble the simulated ones across the frequency range of interest. Though the minimum depths of the return losses are not as pronounced as the simulated counterparts, they remain less than −10 dB across the entire operating bandwidth, indicating good matching for the corresponding ports of this path. The slight discrepancy between the simulated and measured results can be attributed to manufacturing tolerances and measurement errors. Figure 9 also shows that both paths’ measured insertion loss (S_21_ and S_43_) is approximately 1 dB lower than the simulation. This difference is expected due to fabrication imperfections, parasitic effects, and discontinuities in the measurement setup. Finally, from Figure 9, it can also be observed that the device illustrates sound isolation between the crossing paths, S_31_ and S_13_, of approximately −18 dB over the entire frequency span, which broadly matches the simulation results.

**Figure 10:**
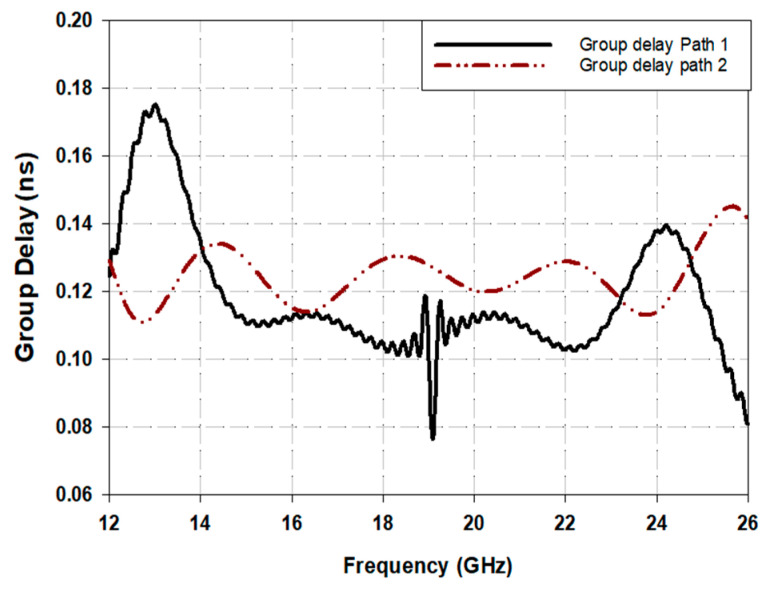
Group delay of the two paths of the proposed crossover.

Figure 10 illustrates the group delays for both crossing paths in the structure, revealing a slight difference in signal timing properties. Path 1 encounters a delay of 0.11 ns, while Path 2 encounters 0.13 ns, across the passband frequency range from 13 GHz to 22 GHz, attributed to Path 1 being electrically shorter. Path 1 follows a direct trajectory over the substrate’s top side, while Path 2 involves a vertical transition, resulting in a difference in group delays. Achieving zero difference in group delays is crucial in certain applications, like Butler matrix phase shifters. Our design nearly meets this requirement, with a group delay difference of less than 0.02 ns, significantly smaller than reported in the literature.

In summary, the measured and simulated results of the proposed crossover are presented, where advantages are highlighted by contrasting its properties with those of the most recently published structures, as depicted in Table 2. It is evident that the proposed crossover is characterized by a wider fractional bandwidth. Furthermore, the difference in group delay between the crossed paths is extremely small, at only 0.02 ns. On the other hand, it is via-less, with an actual size of 6.5 × 7.6 mm^2^.

## 5. Conclusions

A wideband mmWave via-less crossover has been designed, fabricated, and measured. The proposed structure exhibits broadband characteristics of 11 GHz. The key advantage of this design is the via-less feature, coupled with a very low group delay of less than 0.02 ns between the two crossed lines. This enhances its suitability for the Butler matrix used in mmWave phased array feeding networks for remote sensing applications. Two microstrip line transitions were utilized at crossing junctions to achieve these advantages: a stair-shaped microstrip line-to-CPW transition for one line and a vertically coupled hourglass-shaped microstrip-to-similar-CPW transition for the other. Furthermore, the design offers good insertion loss of about −1 dB and isolation of −18 dB across the desired frequency range, making it a unique via-less design with excellent transmission characteristics in the mmWave frequency band.

## Figures and Tables

**Table 1:** Dimension details of the proposed crossover structure.

Structure Part	Type	Corresponding Dimensions (mm)	
L_ex_	MST	Lex	4.34
		Wex	0.5
L_m1_	MST	Lm1	1.16
		Wm1	1.17
L_c1_	GPCW	2wc1	0.653
		Sc1	0.35
		2wc1	0.653
L_c2_	GPCW	Lc2	0.96
		2wc2	0.536
		Sc2	0.144
L_m2_	MST	Lm2	1.66
		Wm2	0.658
W_p_	Coupled MST-Patch	Wp	1.02
		Lp	1.96
W_cp_	Coupled CPW-Patch	Wcp	1.86
		Lcp	1.02
		Sg	0.5
L_GL_	CPW	L_GL_	3.33
		W_GL_	0.5
		S_GL_	0.225

**Table 2:** Comparison of this work with previous proposed designs.

Proposed Design	Actual Size (mm^2^)	Bandwidth (GHz)	Via-Less	Multilayer Profile	Center Frequency (GHz)	Isolation (dB)
[15]	10 × 10	10	No	No	5	23
[13]	10 × 10	10	No	No	5	25
[16]	10 × 20	6	No	No	3	15
[1]	11.3 × 11.3	6 (based on 20 dB RL)	No	No	3	20
[14]	3 × 3	40	No	No	20	19
[8]	8 × 15	8	Yes	No	7	15
[17]	27 × 13	3	No	Yes	10.5	17.3
[18]	24 × 7.11	7	Yes	Non Planar	34.8	20
[19]	10 × 10	6	No	Yes	30	15
This Work	6.5 × 7.6	11	Yes	No	18	15

## Data Availability

The raw data supporting the conclusions of this article will be made available by the authors on request.
